# Long-Term Outcomes of Veterans With a Diagnosis of Heart Failure After COVID-19

**DOI:** 10.1016/j.jacadv.2023.100381

**Published:** 2023-06-07

**Authors:** Vishal Khetpal, Julia Berkowitz, Lan Jiang, Anupama Menon, Nishant Shah, Daithi S. Heffernan, Gaurav Choudhary, James L. Rudolph, Wen-Chih Wu, Sebhat Erqou

Long-term clinical sequelae have been increasingly recognized among individuals after acute COVID-19.^1^ These sequelae may be more pronounced in heart failure (HF) patients, who experience higher rates of COVID-19 hospitalization and in-hospital mortality compared to non-HF patients.^2^

In this retrospective cohort study using US Veteran Health Administration data, we investigated long-term outcomes of hospitalization and mortality among HF patients after COVID-19 diagnosis beyond the acute phase of the disease. Veteran Health Administration data were accessed via the VA Informatics and Computing Infrastructure. The study has received approval from the Providence VA Medical Center Institutional Review Board. Veterans with pre-existing HF (n = 244,302) were identified based on having at least 1 medical encounter in the VA with an International Classification of Disease (ICD) diagnostic code for HF within the year prior to the beginning of the pandemic in the United States (February 2020). COVID-19 cases were identified among the cohort of veterans with pre-existing HF based on a record of a positive severe acute respiratory syndrome-coronavirus-2 (SARS-CoV-2) polymerase chain reaction or antigen test on an outpatient or inpatient nasopharyngeal swab conducted within or reported to the VA between February 1, 2020, and July 31, 2021. COVID-19 cases were age-, sex-, and race-matched with up to 5 veterans with pre-existing HF and no known positive SARS-CoV-2 test during the study period. HF phenotype based on ejection fraction (EF), ie, preserved EF (EF ≥45%, HFpEF) or reduced EF (EF <45%, HFrEF), was determined in a subset (81%) where echocardiogram data were available within the preceding 2 years. Comorbid conditions were defined by ICD codes. The primary outcome was time to all-cause mortality, and the secondary outcome was time to first hospital admission (all causes). Patients were followed from the date of positive SARS-CoV-2 test, ie, time zero (T0), until death or censoring on July 31, 2021. For the controls, we assigned a T0 for each individual based on the distribution of the dates of T0 in the cases to make the follow-up time comparable. Cox-regression models were used to estimate adjusted HRs and 95% CIs, with progressive adjustment for potential confounders. A competing risk model was used for hospital admission outcomes. Analyses were performed using SAS 9.4 (SAS Institute Inc).

The study involved 74,678 veterans with HF (age 72.1 ± 10.2 years, 2.4% female, 71.3% White), including 13,722 veterans with COVID-19 and 60,956 age-, sex-, and race-matched controls without known COVID-19. Twenty-five percent of the cases were already admitted to the hospital (not necessarily for COVID-19 symptoms) at the time of positive SARS-CoV-2 test, ie, at time zero, T0. Similarly, 0.7% of controls were inpatients at T0. Those with COVID-19 had a higher prevalence of hypertension (90.3% vs 85.6%), diabetes mellitus (61.3% vs 52.7%), myocardial infarction (60.9% vs 54.9%), obesity (31.6% vs 25.5%), chronic kidney disease (40.1% vs 30.7%), chronic obstructive pulmonary disease (42.2% vs 35.4%), depression (32.3% vs 25.3%), drug use (6.3% vs 5.7%), and homelessness (3.9% vs 3.1%). Participants were followed for a mean duration of 234 ± 117 days since the positive COVID test. Veterans with HF and COVID-19 had significantly higher risk of mortality (24.3% vs 9.5%) and hospital admissions (28.3% vs 13.7%), as well as 30-day, 90-day, and 180-day event rates compared to controls. Compared to the controls, the COVID-19 group had adjusted HRs of 2.68 (95% CI: 2.56-2.80) for mortality and 1.94 (95% CI: 1.86-2.03) for hospital admissions over the study period. The corresponding adjusted HRs were 1.46 (95% CI: 1.37-1.55) for mortality and 1.33 (95% CI: 1.25-1.40) for hospital admissions, excluding events occurring within the first 30 days of diagnosis. Adjusted HRs were 1.18 (95% CI: 1.10-1.28) for mortality and 1.28 (95% CI: 1.19-1.38) for hospital admissions, excluding events occurring within the first 90 days of infection. Findings were similar between HF patients with preserved or reduced EF (Table 1).

**Table 1:** Hazard Ratios of Mortality and Hospital Admissions During the Study Period (February 1, 2020-July 31, 2021)

Adjustment	Follow-Up From COVID-19 Diagnosis to July 31, 2021, or Censoring				Excluding Events Within First 30 d of COVID-19				Excluding Events Within First 90 d of COVID-19			
	Mortality		Hospital Admission		Mortality		Hospital Admission		Mortality		Hospital Admission	
	HR	(95% CI)	HR	(95% CI)	HR	(95% CI)	HR	(95% CI)	HR	(95% CI)	HR	(95% CI)
Model 1												
Overall^a^	3.08	(2.95-3.21)	2.52	(2.42-2.63)	1.69	(1.59-1.79)	1.72	(1.62-1.82)	1.36	(1.26-1.47)	1.62	(1.51-1.75)
LVEF ≥45%^b^	1.79	(1.64-1.96)	2.26	(2.13-2.40)	1.51	(1.37-1.67)	1.53	(1.42-1.65)	1.39	(1.23-1.58)	1.44	(1.30-1.59)
LVEF <45%^b^	1.68	(1.53-1.84)	2.42	(2.27-2.59)	1.43	(1.28-1.59)	1.68	(1.53-1.83)	1.38	(1.21-1.58)	1.61	(1.44-1.80)
Model 2												
Overall^a^	2.95	(2.82-3.08)	2.25	(2.15-2.35)	1.62	(1.53-1.72)	1.54	(1.45-1.63)	1.31	(1.21-1.41)	1.46	(1.36-1.57)
LVEF ≥45%^b^	1.74	(1.59-1.89)	2.14	(2.02-2.27)	1.46	(1.32-1.62)	1.45	(1.34-1.57)	1.35	(1.19-1.53)	1.37	(1.24-1.52)
LVEF <45%^b^	1.59	(1.44-1.74)	2.29	(2.14-2.45)	1.35	(1.21-1.50)	1.59	(1.45-1.73)	1.31	(1.15-1.51)	1.53	(1.37-1.72)
Model 3												
Overall^a^	2.71	(2.59-2.83)	2.08	(1.99-2.17)	1.48	(1.39-1.57)	1.43	(1.35-1.51)	1.20	(1.11-1.29)	1.37	(1.28-1.48)
LVEF ≥45%^b^	1.57	(1.44-1.72)	1.97	(1.86-2.10)	1.32	(1.20-1.47)	1.33	(1.23-1.44)	1.23	(1.08-1.39)	1.28	(1.16-1.41)
LVEF <45%^b^	1.46	(1.33-1.61)	2.14	(2.00-2.29)	1.25	(1.12-1.39)	1.50	(1.37-1.63)	1.21	(1.06-1.39)	1.46	(1.30-1.64)
Model 4												
Overall^a^	2.68	(2.56-2.80)	1.94	(1.86-2.03)	1.46	(1.37-1.55)	1.33	(1.25-1.40)	1.18	(1.10-1.28)	1.28	(1.19-1.38)
LVEF ≥45%^b^	1.55	(1.42-1.69)	1.84	(1.73-1.95)	1.30	(1.17-1.44)	1.23	(1.41-1.33)	1.22	(1.07-1.38)	1.18	(1.06-1.31)
LVEF <45%^b^	1.43	(1.30-1.58)	2.01	(1.87-2.15)	1.22	(1.09-1.36)	1.40	(1.28-1.53)	1.19	(1.04-1.37)	1.37	(1.22-1.54)

Model 1 = age (matched) + sex (matched) + race (matched) + married status + facility; Model 2 = Model 1 + hypertension, diabetes, chronic kidney disease, myocardial infarction, obesity and EF subtypes; Model 3 = Model 2 + Elixhauser comorbidities + EF subtypes, smoking status, homelessness status; Model 4 = Model 3 + number of ER visits in the previous year + number of hospital admissions in the previous year.

LVEF = left ventricular ejection fraction.

a In the cohort, 18.7% (20.2% with COVID and 12.4% without COVID) were missing EF values. As a result, the average HRs of EF subgroups do not approximate the overall HRs of the cohort. When adjusting for EF subtypes, those with missing EF were included in a separate category, ie, 1 = EF ≥45%, 2 = EF <45%, 3 = EF is missing.

b EF subtype was removed from adjustment for analyses evaluating LVEF ≥45% vs LVEF <45%.

This analysis is among the first to investigate long-term outcomes of HF patients with COVID-19, and suggests, alongside findings from prior studies, that postacute COVID-19 syndrome may be a substantial and enduring disease burden among individuals in this patient population, mediated potentially by increased thrombotic risk, myocardial injury, and arrhythmogenicity in the acute phase of disease.^2^, ^3^, ^4^, ^5^ Unlike some, but not all, prior shorter-term studies of COVID-19 in HF demonstrating increased mortality among those with reduced EF, our outcomes were similar regardless of EF.^3^ Our findings implicate that HF patients who are impacted by COVID-19 infection warrant close clinical surveillance and that aggressive prevention strategies, including vaccination, are needed to reduce the incidence of infection in this patient population.

The limitations of this study merit consideration. First, the generalizability of the findings is limited due to the lack of diversity in a predominantly male veteran population. Second, the study focused on the coronavirus period in which the ‘wild’ and subsequently delta variants were most prevalent among this population (ie, February 2020 to July 2021). While this helps to increase the internal validity of the study, it may not generalize to the omicron variant, which may require a separate study. Third, compared to a control population comprised of individuals not known to have tested positive for SARS-CoV-2 during the study period, individuals who underwent testing, particularly in earlier stages of the pandemic characterized by limited testing availability, may be more likely to have certain characteristics that put them at higher risk for poorer outcomes or were tested while seeking high-acuity care, which could result in confounding by testing indication. This may be particularly important among the COVID-19 cases given the known fact that SARS-CoV-2 infection can be asymptomatic. Hence, a given proportion of the COVID-19 cases are likely to be incidental, discovered due to healthcare contact for other illnesses, biasing the association away from the null. The use of controls that tested negative for COVID-19, on the other hand, may also be fraught with bias due to the “blanket policy” of testing patients who encounter healthcare system for COVID-19. Therefore, the morbidity of controls selected on the basis of negative SARS-CoV-2 test is likely to be significantly higher than that of the true counterfactual cohort, as those patients with higher healthcare contact are likely to be sicker in general (eg, those being admitted for non-COVID-related reasons), biasing the association towards the null. Overall, that the associations observed in the current study using population controls remained moderately strong and significant after extensive adjustment for comorbidity and other potential confounders lends confidence to the findings.

In conclusion, among 74,678 veterans with HF, we found that COVID-19 was associated with significantly increased long-term hospitalization and mortality beyond the first 30 or 90 days of diagnosis, irrespective of HF type. Future studies with a more diverse population, longer follow-up, including the period of the omicron variant, and using multiple different types of comparator groups (ie, population, SARS-CoV-2 negative, historical) can help to further characterize the burden of postacute COVID-19 syndrome among HF patients and identify optimal management strategies for this population.

## References

[1] Nalbandian A., Sehgal K., Gupta A. (2021). Post-acute COVID-19 syndrome. Nat Med.

[2] Ramadan M.S., Bertolino L., Zampino R., Durante-Mangoni E., Monaldi Hospital Cardiovascular Infection Study Group (2021). Cardiac sequelae after coronavirus disease 2019 recovery: a systematic review. Clin Microbiol Infect.

[3] Rumery K., Seo A., Jiang L. (2021). Outcomes of coronavirus disease-2019 among veterans with pre-existing diagnosis of heart failure. ESC Heart Fail.

[4] Goyal P., Reshetnyak E., Khan S. (2021). Clinical characteristics and outcomes of adults with a history of heart failure hospitalized for COVID-19. Circ Heart Fail.

[5] Al-Aly Z., Xie Y., Bowe B. (2021). High-dimensional characterization of post-acute sequelae of COVID-19. Nature.

